# Estimated Association Between Organ Availability and Presumed Consent in Solid Organ Transplant

**DOI:** 10.1001/jamanetworkopen.2019.12431

**Published:** 2019-10-02

**Authors:** Luke J. DeRoos, Wesley J. Marrero, Elliot B. Tapper, Christopher J. Sonnenday, Mariel S. Lavieri, David W. Hutton, Neehar D. Parikh

**Affiliations:** 1 Industrial and Operations Engineering, University of Michigan, Ann Arbor; 2 Division of Gastroenterology and Hepatology, University of Michigan, Ann Arbor; 3 Department of Surgery, University of Michigan, Ann Arbor; 4 School of Public Health, University of Michigan, Ann Arbor

## Abstract

**Importance:**

Presumed consent, or an opt-out organ transplant policy, has been adopted by many countries worldwide to increase organ donation. The implication of such a policy for transplants in the United States is uncertain, however.

**Objective:**

To simulate the potential implications of a presumed consent policy in the United States.

**Design, Setting, and Participants:**

In a decision analytical model, a simulation model was developed using cohort data from January 1, 2004, to December 31, 2014, in the Organ Procurement and Transplantation Network Standard Transplant Analysis and Research files. All US patients (n = 524 359) who were on the waiting list for at least 1 solid organ and all deceased organ donors during the study period were included in the analyses. All data and statistical analyses were performed from January 30, 2019, to July 31, 2019.

**Main Outcomes and Measures:**

Increase in the organs available for donation and life-years gained associated with a 5%, 15%, or 25% increase in deceased donors, based on the published changes from a presumed consent policy.

**Results:**

This study considered 524 359 unique candidates (aged ≥18 years; 320 908 [61.2%] male) for a solid organ transplant from January 1, 2004, to December 31, 2014. With a base case scenario of a 5% presumed consent–associated increase in donors, the removals (owing to death or illness) from the waiting list for all organs would have an associated 3.2% to 10.4% mean reduction, depending on the random or ideal allocation of new organs to patients on the waiting list. Sensitivity analyses showed that waiting list removals could be decreased up to 52%; however, this reduction was not enough to completely eliminate waiting list removals during the study period. The biggest estimated increases in annual life-years gained associated with a presumed consent policy were in kidney transplant candidates (95% CIs by deceased donor increase: 5% increase, 3440-3466 years; 15% increase, 10 321-10 399 years; 25% increase, 17 201-17 332 years) and liver transplant candidates (95% CIs by deceased donor increase: 5% increase, 898-905 years; 15% increase, 2693-2714 years; 25% increase, 4448-4523 years). Adoption of a presumed consent policy could result in a 4295-year (95% CI, 4277-4313 years) to 11 387-year (95% CI, 11 339-11 435 years) increase in life-years, accounting for the survival advantages associated with a transplant.

**Conclusions and Relevance:**

In this study, presumed consent was estimated to be associated with modest but important improvement in the number of organ transplants and increases in life-years gained for patients awaiting an organ transplant. Further consideration and even debate about the ethical and public policy implications of a presumed consent policy are warranted.

## Key Points

**Question:**

What are the plausible implications of a presumed consent transplant policy for waiting list outcomes in the United States?

**Findings:**

In this simulation study of 524 359 potential organ recipients in a decision analytical model, a presumed consent policy was estimated to be associated with a reduction in waiting list removals. This estimation translated to an increase in life-years gained for patients in this simulation.

**Meaning:**

This study suggests that implementation of a presumed consent policy could be the most immediate way to expand organ donation, although presumed consent alone is not likely to solve organ shortages in the United States.

## Teaser

This decision analytical model study uses a simulation model to evaluate the potential implications of a presumed consent policy for deceased organ donation allocation and availability in the United States.

## Introduction

Organ transplant is a life-saving and cost-effective intervention for patients with organ failure.^1^ However, in the United States, the supply of organs cannot meet the need of patients on the waiting list, resulting in prolonged morbidity and higher mortality for these patients.^2^ The US organ transplant waiting list continues to grow yearly despite recent increases in the number of deceased donors, and approximately 7500 patients die yearly while awaiting an organ transplant.^3^ In general, the number of available donors can be enlarged in 2 ways: (1) expand the donor pool and (2) optimize donor yield. Current mechanisms for expanding the donor pool include increasing donor registrations, using extended criteria donors, and promoting living donor transplant. Optimizing the donor yield requires the use of best practices and emerging technologies (eg, machine perfusion) in donor consent and organ retrieval.^4,5,6^ Even with these mechanisms, the donor pool is limited and will likely shrink in coming years because of changing US demographics and population health (eg, obesity prevalence, aging population), further exacerbating the disparity between donors and recipients.^7,8^

A potential, albeit controversial, policy that has been proposed to increase organ donation is presumed consent or opting out.^9^ Such a policy would make willingness to donate the default option unless an individual explicitly opts not to be an organ donor.^10^ The controversy surrounding presumed consent stems from the loss of patient autonomy and fear that such a system would decrease organ donation.^11^ However, variations of this policy have been adopted in several other countries and have yielded mixed results.^12,13,14,15,16^ Nonetheless, in the United States, a suboptimal 54% of adults are registered organ donors, and evidence suggests that lack of consent plays a role in preventing a donation from 20% to 40% of otherwise eligible deceased donors.^17,18^ Thus, by reducing the number of potential donors who do not donate because of lack of authorization, presumed consent has been proposed as an avenue to increase organs available for transplant. To estimate the associated implications of a presumed consent policy in the United States, we developed a model that examined such a policy in a historical cohort of wait-listed patients.

## Methods

In this study, we constructed a waiting list simulation model to estimate the implications of presumed consent for US patients on a national transplant waiting list from January 1, 2004, to December 31, 2014. We used a range of values to simulate the association between the increase of such a policy and donation rates, according to existing literature.^9^ We followed the Consolidated Health Economic Evaluation Reporting Standards (CHEERS) reporting guidelines for decision analytical model studies.^19^ The University of Michigan Institutional Review Board approved this study and waived the requirement to obtain informed consent because the study used only deidentified data.

### Data Sources

We obtained data from January 1, 2004, to December 31, 2014, included in the Organ Procurement and Transplantation Network Standard Transplant Analysis and Research (STAR) files to develop a waiting list model for solid organ transplant. All individual-level and hospital-level data were deidentified prior to our receipt of these STAR files, and encrypted patient identification numbers were used for our analysis.

Individual-level data were restricted to adult candidates (aged ≥18 years) for solid organ donation (heart, kidney, liver, lung, or pancreas) and included age, organ(s) requested, date added to the waiting list, date removed from the waiting list, and removal reason. Removal reasons were categorized as follows: (1) received living donor allograft, (2) received deceased donor allograft, (3) died while waiting for transplant or became too ill to undergo transplant, and (4) other.

We analyzed the STAR files on deceased organ donors to estimate organ yield rates per donor. Data included donor age and the number of organs successfully transplanted from each adult deceased organ donor during the study period.

### Waiting List Model

We simulated the association between a presumed consent policy and waiting list outcomes by organ type in monthly intervals during the 11-year study period (inclusive of the entirety of 2014). The key metrics used to assess the implications of a presumed consent policy were the number of patients on the waiting list and the number of patient removals owing to death or illness. To calculate these metrics, we used a queuing model with deterministic service and arrival times for each patient based on historical patient data. The model simulated the changes associated with presumed consent by increasing the number of deceased donors who donated at least 1 organ and then adjusting waiting list additions and removals accordingly.

For analyses that considered all organs, candidates awaiting multiple organs were listed only once (eTable in the Supplement). For analyses scoped to individual organs, candidates awaiting multiple organs were listed on all corresponding waiting lists. In all instances, candidates simultaneously listed at multiple centers for the same organ were counted only once. As new organs became available, the model considered 2 allocation policies when adjusting waiting list removal rates. Candidates who were listed, underwent a transplant, and were later relisted were counted multiple times. A schematic of the model is included in eFigure 1 in the Supplement.

A systematic review by Rithalia et al^9^ examining the association between the institution of a presumed consent policy and deceased donor availability from several countries showed that the policy was associated with up to a 25% increase in deceased donor availability. However, more recent studies have reported more modest improvement in deceased donor availability.^14,20,21^ Accordingly, we used a model with a practical range of 5% to 25% potential deceased donor increases with such a policy change in the United States. A 5% increase in deceased organ donations was the base case for a presumed consent policy. In all cases, the living donor donations and waiting list addition quantities were not changed from true historical values.

To translate a deceased donor increase to an increase in the number of transplants, we incorporated organ yield rates into the model. Because organ yield rates can vary, the model considered a nonparametric 95% CI for these yield estimates in addition to the mean values.

In simulating the availability of additional donors, substantial variation in the allocation of organs to patients can be observed.^22^ Deciding which individual will receive a newly available allograft depends on several factors, such as candidate blood type, geography, physician decision-making, and candidate availability. Limitations in this data set and modeling methods prevented us from using a single prescriptive allocation method that appropriately matched clinical practice. As a result, we developed 2 allocation algorithms to represent the range of potential outcomes. This range described the primary uncertainty in the model’s estimates. We assumed that the true expected outcomes of a presumed consent policy would fall somewhere within this range.

In the first allocation algorithm, random allocation, additional organs were assigned to currently waiting candidates regardless of demographics such as age, time on the waiting list, medical illness, or waiting list priority. Given that current clinical allocation policies are designed to ideally distribute organs to patients with the most need, random allocation would serve as a lower bound for the possible implication of presumed consent.

In the second allocation algorithm, ideal allocation, the model matched additional organs with candidates who would have otherwise been removed from the list because of death or because they were medically unsuitable for a transplant. Patients who would have been delisted because of death or illness during that period were given newly available organs first, just before their removal from the waiting list. This scenario was designed to emulate the case in which clinicians are able to perfectly forecast patient health and need and in which all available organs are compatible with the sickest patients. This scenario is considered ideal for patient health and, although unlikely, can serve as an upper bound for the possible advantages of presumed consent. The true implication of a presumed consent policy would lie somewhere between a random and an ideal allocation.

The model output included the estimated number of waiting list candidates in each year during the study period and the number of candidates in each removal category for each organ. This output was combined with published results on organ transplant survival to estimate the potential implications of presumed consent for patient life-years gained from transplant.^23^

### Statistical Analysis 

All data and statistical analyses were performed from January 30, 2019, to July 31, 2019, using R, version 3.5.1 (R Project for Statistical Computing).^24^ The uncertainty in the model’s estimates came from 3 primary sources: (1) variation in the increase in deceased donors, (2) variation in the allocation of additional organs to waiting candidates, and (3) variation in organ yield for additional donors.

We estimated the uncertainty in the donor increase by calculating a range of outcomes given a deterministic donor increase. We estimated the uncertainty in organ allocation by developing 2 allocation algorithms and calculating the confidence intervals in organ yield. We estimated the uncertainty in organ yield by using deceased donor information from the STAR files. To calculate the mean yield for each organ, we performed bootstrapping on individual donor data from 2004 to 2014 using 1000 replications. We used the empirical distribution of results to generate a 95% CI for each mean.

## Results

### Historical Data

From January 1, 2004, to December 31, 2014, a total of 524 359 unique potential organ recipients (aged ≥18 years; 320 908 [61.2%] male) were on the waiting list. The number of wait-listed candidates on a year-to-year basis widely fluctuated by organ type, from an increase of 45 373 kidney transplant candidates (82.8%) to a decrease of 2036 lung transplant candidates (55.9%). The number of patients on the waiting list at the beginning of each year is shown in the eTable in the Supplement. The mean total monthly number of removals from the waiting list owing to death or illness was 848 for solid organ transplant during the study period. Removals by organ type included 41 (4.8%) for heart transplant, 536 (63.2%) for kidney, 212 (25.0%) for liver, 32 (3.8%) for lung, and 27 (3.2%) for pancreas.

By bootstrapping deceased donor data, we found that the 95% CIs for the mean organ yield for deceased donors were as follows: 0.289 to 0.292 heart per donor, 1.465 to 1.476 kidneys per donor, 0.765 to 0.770 liver per donor, 0.340 to 0.349 lung per donor, and 0.155 to 0.160 pancreas per donor over the model period.^25^

### Presumed Consent Policy

**Figure 1.**
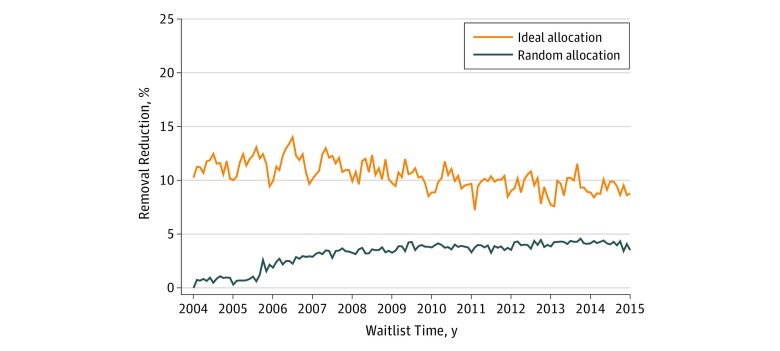
Expected Overall Candidate Removals From the Waiting List, 2004-2014 Removals owing to death or illness were associated with a 5% increase in donors with presumed consent under random and ideal allocation. Shaded areas represent a nonparametric 95% CI for estimates based on variation in organ yield.

We considered a range of increases to the number of deceased organ donors associated with the implementation of a presumed consent policy. Figure 1 presents the potential association of the presumed consent base case (5% increase in donation) with the number of candidates removed owing to death or illness under both allocation approaches. The association with the number of removals varied between scenarios. In the base case, random allocation was associated with a mean reduction of 26.8 removals (3.2%) owing to death or illness over the study period. Ideal allocation was associated with a mean reduction of 84.2 removals (10.4%).

The model suggested that ideal allocation would be associated with an immediate change in the mean number of removals owing to death or illness (77.4 [11.3%] in 2004). This removal percentage trended lower over time (88.8 [9.2%] in 2014) because the increase in donors was not enough to completely eliminate removals, so the waiting list continued to grow over the study period (from 78 011 candidates in 2004 to 119 989 candidates at the end of 2014). Conversely, random allocation initially was associated with a smaller change in removals (5.0 [0.7%] in 2004), which grew over the study period (to 40.1 [4.1%] in 2014). This change occurred because, with random allocation, the reduction in the rate of death or illness was proportional to the size of the waiting list. Random allocation reduced not only the size of the waiting list but also the number of removals (from 78 011 candidates in 2004 to 115 105 candidates at the end of 2014).

**Figure 2.**
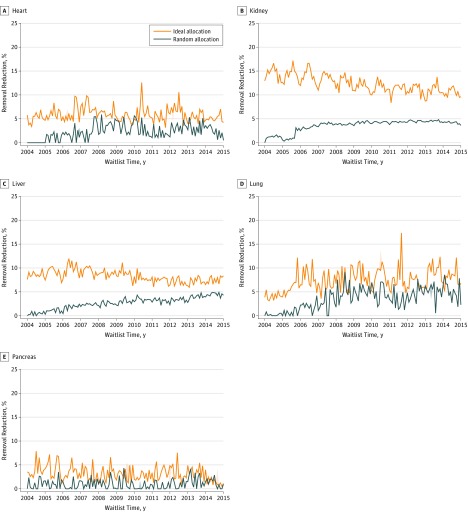
Expected Candidate Removals From the Waiting List by Organ, 2004-2014 Removals owing to death or illness were associated with a 5% increase in donors with presumed consent under random and ideal allocation for the following organs: heart (A), kidney (B), liver (C), lung (D), and pancreas (E). Shaded areas represent a nonparametric 95% CI for estimates based on variation in organ yield.

A breakdown of the change over time in the number of removals owing to death or illness by organ type and allocation policy is presented in Figure 2 and eFigure 2 in the Supplement. The temporal variation in the curves primarily reflects the variation in the historical monthly removals from the waiting list. Table 1 shows the 95% CIs for the estimated mean monthly reduction in these removals for each organ over a range of presumed consent–associated donation increases. In the simulation, even assuming an ideal allocation scenario with a 25% increase in presumed consent, not enough donors were generated to eliminate deaths or removals owing to illness while candidates awaited an organ transplant (95% CIs for mean monthly reduction by organ: heart, 29.42-29.96; kidney: 61.52-61.99; liver, 41.35-41.71; lung: 36.41-37.43; pancreas: 16.36-16.86).

**Table 1.** Mean Monthly Reduction in Waiting List Removals by Organ, 2004-2014

Allocation Estimate^a^	Heart Transplant	Kidney Transplant	Liver Transplant	Lung Transplant	Pancreas Transplant	Combined Estimate^b^
Mean monthly removals owing to death or illness, No.						
Historic allocation	41.42	536.24	212.46	32.30	27.15	816.66
Mean monthly % of reduction in removals owing to death or illness, 95% CI						
5% Presumed consent–associated donation increase^c^						
Random allocation	2.29-2.33	3.58-3.60	2.66-2.67	3.50-3.52	0.98-1.00	3.17-3.19
Ideal allocation	5.82-5.90	12.31-12.40	8.28-8.33	7.35-7.42	3.14-3.28	10.42-10.49
15% Presumed consent–associated donation increase^c^						
Random allocation	6.69-6.84	10.21-10.28	9.46-9.56	9.59-9.81	2.37-2.37	9.57-9.65
Ideal allocation	17.63-17.96	36.92-37.19	24.82-25.02	21.87-22.50	9.80-10.05	31.25-31.52
25% Presumed consent–associated donation increase^c^						
Random allocation	11.06-11.29	16.84-16.97	16.26-16.40	15.71-16.20	4.50-4.68	15.97-16.12
Ideal allocation	29.42-29.96	61.52-61.99	41.35-41.71	36.41-37.43	16.36-16.86	52.09-52.54

^a^ Random and ideal allocation estimates were assumed to be the lower and upper bounds, respectively, for a practical allocation policy. The true mean percentage of reduction in removals was expected to be between these estimates. The ranges represent nonparametric 95% CIs of the policy estimates owing to variation in organ yield.

^b^ Candidates on multiple waiting lists were counted only once.

^c^ Percentages of presumed consent referred to the percentage increase in deceased organ donation and corresponding model adjustment.

The number of candidates on the waiting list by allocation policy for the base case is shown in eFigure 3 in the Supplement. Under ideal allocation, presumed consent would need to generate enough additional organs to eliminate removals owing to death or illness before affecting the other candidates who would remain on the waiting list. The model’s estimated association of presumed consent with the number of waiting list candidates is shown in Table 2 and eFigure 4 in the Supplement. Random allocation in the base case scenario was associated with a reduction in waiting list quantities (95% CIs for reduction in waiting list growth from January 1, 2004, to December 31, 2015, by organ: heart, 2.35-2.41; kidney, 6.89-6.94; liver, 4.89-4.94; lung, 2.02-2.04; pancreas, 0.72-0.75). However, this outcome occurred because the algorithm does not perfectly assign additional organs to patients who would have died or become too sick. Random allocation was associated with an estimated 4% reduction in the number of candidates awaiting solid organ transplant at the end of the study period given a 5% increase in presumed consent, 14% reduction in candidates given a 15% increase in presumed consent, and 23% reduction in candidates given a 25% increase in presumed consent.

**Table 2.** Change in Number of Waiting List Candidates, 2004-2014

Allocation Estimate^a^	Heart Transplant	Kidney Transplant	Liver Transplant	Lung Transplant	Pancreas Transplant	Combined Estimate
Opt-in policy, % change in candidates						
Historical allocation	10.79	82.83	(3.65)^b^	(55.92)^b^	(21.28)^b^	53.81
Presumed consent estimates, % change in candidates, 95% CI						
5% Presumed consent–associated donation increase^c^						
Random allocation	8.38-8.44	75.89-75.94	(8.59)-(8.54)^b^	(57.96)-(57.94)^b^	(22.03)-(22.00)^b^	47.85-47.91
Ideal allocation	10.79-10.79	82.83-82.83	(3.65)-(3.65)^b^	(55.92)- (55.92)^b^	(21.28)- (21.28)^b^	53.81-53.81
15% Presumed consent–associated donation increase^c^						
Random allocation	2.54-2.67	58.73-58.92	(17.34)-(17.24)^b^	(63.04)-(62.88)^b^	(24.32)-(24.18)^b^	33.84-34.01
Ideal allocation	10.79-10.79	82.83-82.83	(3.65)-(3.65)^b^	(55.92)- (55.92)^b^	(21.28)- (21.28)^b^	53.81-53.81
25% Presumed consent–associated donation increase						
Random allocation	(3.56)-(3.31)^b^	41.47-41.82	(26.15)-(25.95)^b^	(67.58)-(67.33)^b^	(26.97)-(26.80)^b^	19.76-20.06
Ideal allocation	10.79-10.79	82.83-82.83	(3.65)-(3.65)^b^	(55.92)- (55.92)^b^	(21.28)- (21.28)^b^	53.81-53.81
Presumed consent–associated donation increase required to eliminate waiting list over study period, 95% CI^d^						
Random allocation	203.39-207.54	105.70-106.50	107.71-108.55	94.45-97.00	350.66-361.29	350.66-361.29
Ideal allocation	206.78-211.00	115.18-116.06	119.39-120.32	111.62-114.64	388.23-400.00	388.23-400.00

^a^ Random and ideal allocation estimates were assumed to be lower and upper bounds, respectively, for a practical allocation policy. The true annual increase in life-years gained was expected to be between these allocation estimates. The ranges in this table represent a nonparametric 95% CI of the policy estimates owing to variation in organ yield.

^b^ Values in parentheses represent percentage decreases.

^c^ Percentages of presumed consent referred to the percentage increase in deceased organ donation and corresponding model adjustment.

^d^ Eliminating the waiting list over the study period was defined as having no candidates on the waiting list on December 31, 2014, after accounting for all monthly waiting list additions and removals.

The model under random allocation had an associated reduction in the combined waiting list growth, from 53.81% to 47.85% in the base case. The simulation revealed that the estimated deceased donor increase required to eliminate organ waiting lists in the study period varied among organs and between policies (estimated percentage range of deceased donor increase required by organ: heart, 203.4%-211.0%; kidney, 105.7%-116.1%; liver, 107.7%-120.3%; lung, 94.5%-114.6%; pancreas, 350.7%-400.0%). This required increase ranged from 94.45% for the lung waiting list under random allocation to 400.00% for the pancreas waiting list under ideal allocation. Under ideal allocation, using the assumed 25% upper bound on the increase in deceased organ donors, the model estimated no change in the quantity of wait-listed candidates. This occurred because, in the ideal allocation algorithm, all excess organs were distributed to patients who would have otherwise left the waiting list, and the estimated net number of waiting patients remained the same. Consequently, we omitted graphics that demonstrated the number of wait-listed candidates under ideal allocation. eFigure 5 in the Supplement shows the change by organ type under random allocation.

Sensitivity analyses showed that waiting list removals could be decreased up to 52%; however, this reduction was not enough to completely eliminate waiting list removals during the study period.

### Survival Advantage

Using the estimated survival advantage associated with organ transplant, Table 3 shows the expected life-years gained under varying levels of presumed consent–associated donor increase with a random or an ideal allocation. The model estimated that, over the study period, the presumed consent base case would have been associated with an annual mean gain of approximately 4000 life-years under random allocation. The biggest estimated increases in annual life-years gained associated with a presumed consent policy were in kidney transplant candidates (95% CIs by deceased donor increase: 5% increase, 3440-3466 years; 15% increase, 10 321-10 399 years; 25% increase, 17 201-17 332 years) and liver transplant candidates (95% CIs by deceased donor increase: 5% increase, 898-905 years; 15% increase, 2693-2714 years; 25% increase, 4448-4523 years). With a 25% donor increase under random allocation, the mean annual life-years gained for all organs was up to 21 565. Under ideal allocation, the mean annual life-years gained ranged from 11 339 in the base case to 57 173 at higher deceased donor increases. Adoption of a presumed consent policy could result in a 4295-year (95% CI, 4277-4313 years) to 11 387-year (95% CI, 11 339-11 435 years) increase in life-years, accounting for the survival advantages associated with a transplant.

**Table 3.** Mean Annual Estimated LYG by Organ Transplant and Presumed Consent Implication

Allocation Estimate of Annual LYG	95% CI					
	Heart Transplant	Kidney Transplant	Liver Transplant	Lung Transplant	Pancreas Transplant	Combined Estimates^a^
**Random Allocation**						
LYG per transplant^b^	4.9	4.4	4.3	2.6	2.4	NA
5% Presumed consent–associated donation increase	138-141	3440-3466	898-905	69-71	25-25	4277-4313
15% Presumed consent–associated donation increase	415-423	10 321-10 399	2693-2714	208-213	74-76	12 831-12 939
25% Presumed consent–associated donation increase	691-705	17 201-17 332	4488-4523	346-356	123-127	21 385-21 565
**Ideal Allocation**						
LYG per transplant^b^	9.5	12.1	10.1	4.9	14.5	NA
5% Presumed consent–associated donation increase	268-274	9461-9532	2108-2125	131-134	149-153	11 339-11 435
15% Presumed consent–associated donation increase	804-821	28 382-28 597	6324-6374	392-402	447-460	34 018-34 304
25% Presumed consent–associated donation increase	1340-1368	47 303-47 662	10 541-10 623	653-670	745-767	56 697-57 173

Abbreviations: LYG, life-years gained; NA, not applicable.

^a^ Combined estimates were weighted to account for patients who received multiple allografts.

^b^ Life-years gained per transplant were estimated using results from Rana et al.^23^ Standard errors for these estimates were not provided. The random and ideal allocation estimates were assumed to be lower and upper bounds, respectively, for a practical allocation policy. The true annual increase in LYG was expected to be between these allocation estimates. The ranges in this table represent a nonparametric 95% CI of the policy estimates owing to variation in organ yield.

## Discussion

Presumed consent is a policy that has been proposed in several countries; however, the realistic implications of such a policy in the United States are uncertain. In this analysis, we used estimates of the presumed consent–associated changes derived from other countries that have instituted such a policy.^9,14^ We found that, even in the best-case scenario, the presumed consent policy was associated with modest improvements for waiting lists for all organs and would not resolve removals due to death or illness under an ideal allocation algorithm. This finding highlights the multifaceted approach needed even if deceased donation is increased through a policy change such as presumed consent.

### Expectations and Precedents for Presumed Consent

In this analysis, we modeled a 5% to 25% increase in deceased donation and conservatively used 5% as a base case because of the well-established deceased donor transplant system with relatively high donation rates in the United States. The upper bound of a 25% increase was based on the largest systematic review of presumed consent adopted in other countries, including Austria, Belgium, and Singapore, in which the policy was associated with a 20% to 25% increase in the number of donors available.^9^ This range of associated increase with presumed consent was also confirmed in a separate analysis from several European countries that accounted for patient willingness to donate.^16^ Data from before and after policy implementation in several countries in South America and Europe have shown even more dramatic increases in liver and kidney donation rates (28%-1186%), but other policy changes and secular factors may have played a role in the results.^14^ To date, Spain, for example, has the highest deceased donor donation rates in the world and has had a presumed consent policy since 1979.^12^ In contrast, recent data from the early experience in Wales have shown no measurable implication for donor availability 18 months after the policy change, although the policy has not been enforced long enough for measurement of its true implication.^20^ A systematic analysis of the presumed consent policies adopted in several European countries concluded that countries with the largest increase in donors do not seek family consent prior to donation and maintain a central donor registry.^15^ Differences in the specifics of a presumed consent policy appear to have a substantial association with the ultimate deceased donor yield.

Similarly, it is possible that a presumed consent policy would not be associated with measurable donor availability changes if instituted in the United States. This scenario could stem from several issues surrounding the implementation (eg, public backlash with a high proportion of patients opting out) or execution (eg, inadequate ability to scale resources according to donor increase) and could yield no increase or a possible decrease in deceased donor organs available for transplant. The United States has one of the highest donation rates in the world with the current opt-in system, and thus the gains in donors seen in other countries with an opt-out system might not translate to similar gains in the United States.^26^ However, given the gap in registered donors (54% of US adults) and those who profess their support for organ donation (93% of US adults), presumed consent may remove a substantial barrier for donor authorization.^26^

Several factors affect organ donation. Economic conditions, structural changes to the organ transplant infrastructure, health care system characteristics, and societal norms must be taken into account when measuring the consequences of policy changes over time. The mixed results after implementation of presumed consent policy likely reflect the complex interaction of these factors and highlight the difficulty of extrapolating the experience of one country to another. The specifics of a given country’s policy implementation may also be factors in the availability of deceased donors, including the details on how patients opt out and the ultimate role of the potential donor’s family in making organ donation decisions.

### Public Policy Implication

A point of contention about implementation of presumed consent has been public backlash to such a policy. A 2012 survey of 3200 representative US adults addressed presumed consent. The survey found that most (51.1%) of the US residents surveyed supported or strongly supported a presumed consent policy, whereas 23.4% of patients would opt out of such a system.^27^ In addition, early data from the policy implementation in Wales suggest that implementation has not yet met with public backlash.^20^ However, careful planning and public education, with acknowledgment of the core principle of individual autonomy that permeates the US culture, may be required before the implementation of a presumed consent policy to ensure that the risk of backlash is minimized.^26^ Implementation of such a policy could be trialed in a subset of organ procurement organizations, given the uncertain implication of such a policy.

The other potential drawback to a presumed consent policy could be a decrease in living donor transplant, currently a substantial source of kidney transplants in the United States and a growing source of liver transplant. However, as our analysis suggests, presumed consent alone would be unable to counteract the ongoing growth of the waiting list for living donor–eligible patients (kidney and liver); thus, increasing donor availability in other ways, including promoting living donation, would be an ongoing need. To our knowledge, no published data are available as to the magnitude that a presumed consent policy has had on living donations in the countries that have implemented such a policy.

Ultimately, presumed consent alone is not likely to solve organ shortage in the United States. However, it could be associated with large gains in life-years and could be highly cost-effective for the US health care system given the overall cost-effectiveness of solid organ transplant.^28,29,30^

### Strengths and Limitations

This study has many strengths and limitations. First, we used a historical cohort to simulate a presumed consent system, and these results may not necessarily apply to a contemporary cohort of patients. However, the population of patients with organ failure who require a transplant only continues to increase with commensurate growth in waiting list removal; thus, the estimates in this analysis may be conservative.^2^ Other metrics such as organ yield may change with the advent of new technologies (eg, machine perfusion), and thus the overall changes associated with a presumed consent policy on transplants and life-years gained would likely fluctuate. Second, any organ allocation policy changes created for individual organs may have implications for the estimates presented in this analysis. Third, we could not accurately simulate the allocation of newly available organs associated with presumed consent because of the central role in human decision-making in organ allocation; thus, we estimated random allocation to approximate the lower bounds and ideal allocation to approximate the upper bounds. Fourth, we obtained the most appropriate estimate of life-years gained currently available in the literature. However, under random allocation, these estimates are a lower bound for life-years gained, as they are based on already realized gains by patients who underwent transplant. Many of the patients in the study were still alive at the time of analysis from which we derived the estimated advantages and were therefore still accruing life-years.^23^ Thus, these estimates for life-years gained are also likely lower bounds for changes in life-years associated with presumed consent. In the ideal allocation approach, we used mean posttransplant survival because all organs were allocated to patients who would have otherwise been removed from the waiting list.

## Conclusions

In this simulation study, we showed a potential association of increase in organ donation and life-years gained with the implementation of a presumed consent policy in the United States. Although the implementation of presumed consent has ethical concerns, our findings suggest that such a policy could be the most immediate way to expand organ donation. Further public discourse about the ethics, logistics, and risks of a presumed consent policy are appropriate given the potential benefits.

## Notes

Supplement. **eTable.** Waitlist Candidates on January 1 of Each Respective Year **eFigure 1.** Overview of Waitlist Model Dynamics **eFigure 2.** Expected Reduction in Removals From the Waitlist Due to Death or Illness From 2004-2014 Associated With a 5% Increase in Donors With Presumed Consent Using Ideal Allocation **eFigure 3.** Expected Number of Unique Candidates on a Solid-Organ Transplant Waiting List From 2004-2014 Associated With a 5% Increase in Donors With Presumed Consent Using Random and Ideal Allocations **eFigure 4.** Expected Number of Unique Candidates on a Solid-Organ Transplant Waiting List From 2004-2014 Associated With a Range of Increases in Donors With Presumed Consent Using Random Allocation **eFigure 5.** Expected Number of Unique Candidates on a Solid-Organ Transplant Waiting List From 2004-2014 Associated With a 5% Increase in Donors With Presumed Consent Using Random Allocation by Organ: a) Heart; b) Kidney; c) Liver; d) Lung; e) Pancreas
