# Supplementary material for: Estimated Association Between Organ Availability and Presumed Consent in Solid Organ Transplant
Source: JAMA Netw Open. 2019 Oct 2;2(10):e1912431. doi: 10.1001/jamanetworkopen.2019.12431 (PMC6777259; doi:10.1001/jamanetworkopen.2019.12431)
Supplement: Supplement. — eTable. Waitlist Candidates on January 1 of Each Respective Year eFigure 1. Overview of Waitlist Model Dynamics eFigure 2. Expected Reduction in Removals From the Waitlist Due to Death or Illness From 2004-2014 Associated With a 5% Increase in Donors With Presumed Consent Using Ideal Allocation eFigure 3. Expected Number of Unique Candidates on a Solid-Organ Transplant Waiting List From 2004-2014 Associated With a 5% Increase in Donors With Presumed Consent Using Random and Ideal Allocations eFigure 4. Expected Number of Unique Candidates on a Solid-Organ Transplant Waiting List From 2004-2014 Associated With a Range of Increases in Donors With Presumed Consent Using Random Allocation eFigure 5. Expected Number of Unique Candidates on a Solid-Organ Transplant Waiting List From 2004-2014 Associated With a 5% Increase in Donors With Presumed Consent Using Random Allocation by Organ: a) Heart; b) Kidney; c) Liver; d) Lung; e) Pancreas [file jamanetwopen-2-e1912431-s001.pdf]

Supplementary Online Content

DeRoos LJ, Marrero WJ, Tapper EB, et al. Estimated association between organ availability and presumed consent in solid organ transplant. *JAMA Netw Open*. 2019;2(10):e1912431. doi:10.1001/jamanetworkopen.2019.12431

eTable. Waitlist Candidates on January 1 of Each Respective Year

eFigure 1. Overview of Waitlist Model Dynamics

eFigure 2. Expected Reduction in Removals From the Waitlist Due to Death or Illness From 2004-2014 Associated With a 5% Increase in Donors With Presumed Consent Using Ideal Allocation

eFigure 3. Expected Number of Unique Candidates on a Solid-Organ Transplant Waiting List From 2004-2014 Associated With a 5% Increase in Donors With Presumed Consent Using Random and Ideal Allocations

eFigure 4. Expected Number of Unique Candidates on a Solid-Organ Transplant Waiting List From 2004-2014 Associated With a Range of Increases in Donors With Presumed Consent Using Random Allocation

eFigure 5. Expected Number of Unique Candidates on a Solid-Organ Transplant Waiting List From 2004-2014 Associated With a 5% Increase in Donors With Presumed Consent Using Random Allocation by Organ: a) Heart; b) Kidney; c) Liver; d) Lung; e) Pancreas

This supplementary material has been provided by the authors to give readers additional information about their work.

© 2019 DeRoos LJ et al. *JAMA Network Open*

eTable. Waitlist Candidates on January 1 of Each Respective Year

Year	*Heart*	*Kidney*	*Liver*	*Lung*	*Pancreas*	*All Organs*[a]
2004	3,252	54,779	15,249	3,641	3,665	78,011
2005	2,938	58,507	15,409	3,619	3,777	81,623
2006	2,742	63,010	15,447	2,915	3,868	85,254
2007	2,593	67,759	15,490	2,649	3,805	89,612
2008	2,393	72,946	15,331	2,007	3,643	93,677
2009	2,498	76,660	14,919	1,881	3,565	96,828
2010	2,792	81,801	14,967	1,755	3,424	102,038
2011	2,886	86,307	15,301	1,706	3,384	106,729
2012	2,829	89,399	15,295	1,606	3,173	109,514
2013	3,073	93,526	15,077	1,588	3,069	113,449
2014	3,427	97,774	15,106	1,563	2,968	117,914

[a]Candidates listed on multiple waiting lists were only counted

© 2019 DeRoos LJ et al. *JAMA Network Open*

eFigure 1. Overview of Waitlist Model Dynamics

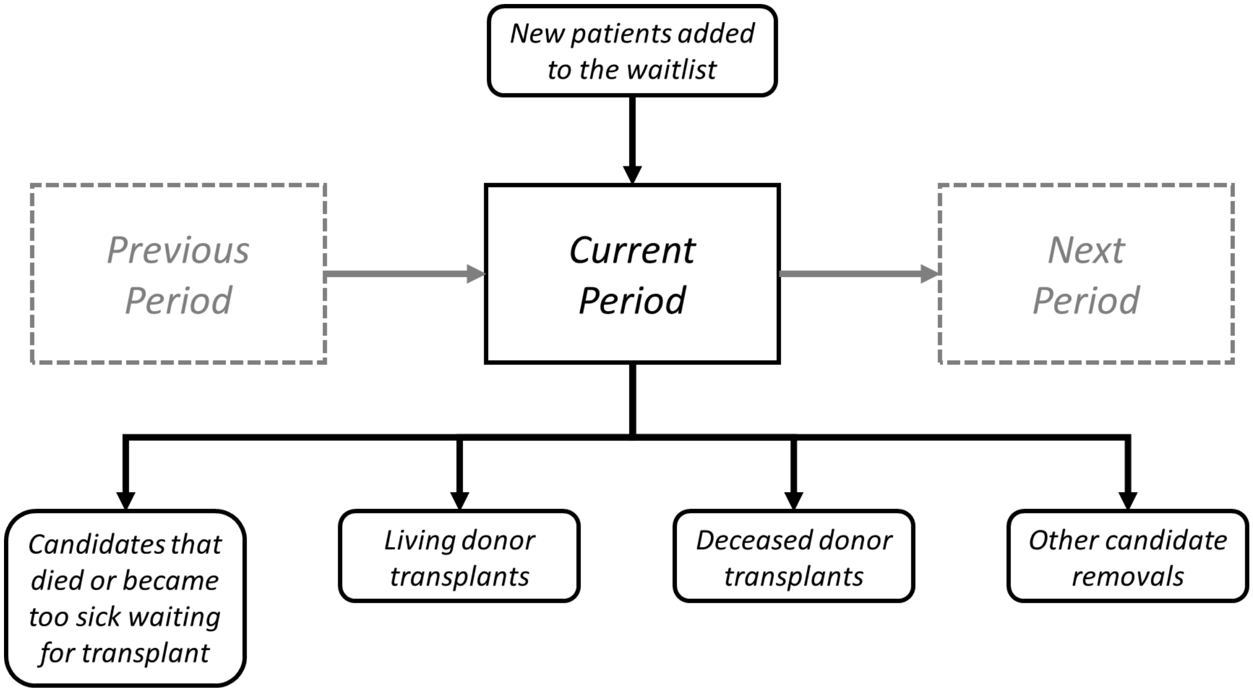

© 2019 DeRoos LJ et al. *JAMA Network Open*

eFigure 2. Expected Reduction in Removals From the Waitlist Due to Death or Illness From 2004-2014 Associated With a 5% Increase in Donors With Presumed Consent Using Ideal Allocation

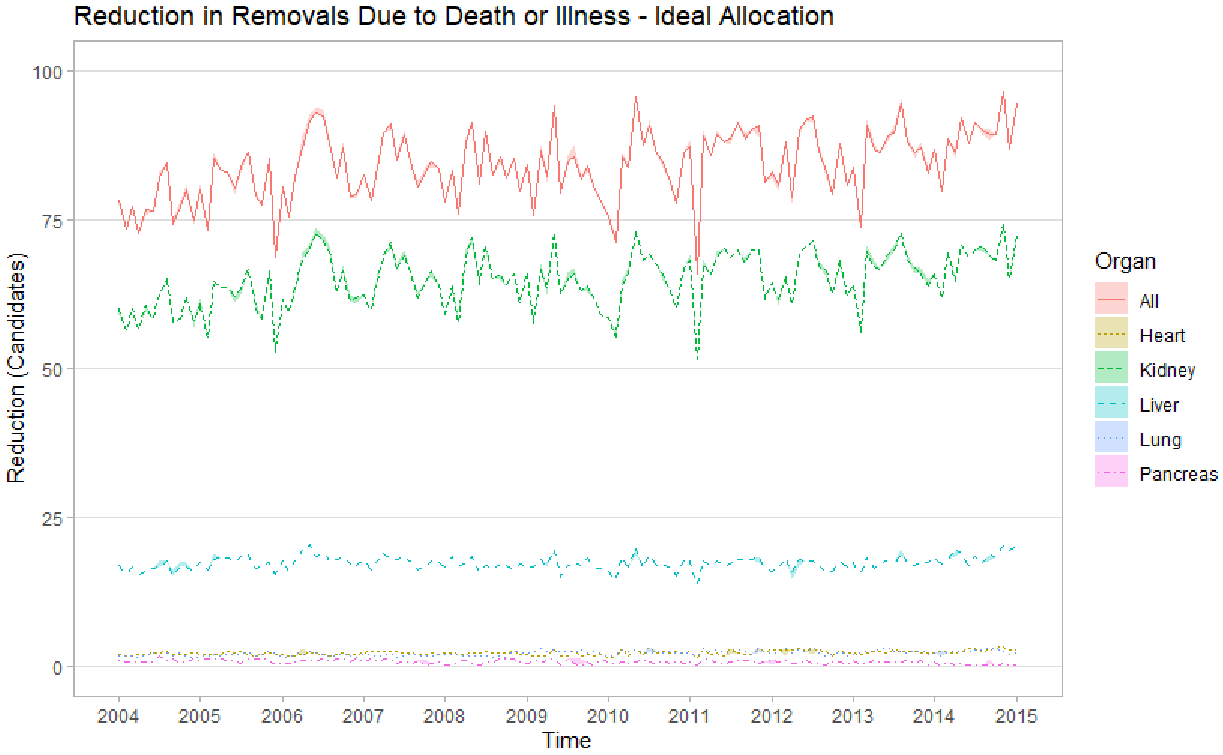

The ideal allocation policy estimates are assumed to be upper bounds for a practical allocation policy. The true reduction in removals due to death or illness is expected to be below these estimates. The ranges represent a non-parametric 95% confidence interval on the policy estimates due to variation in organ yield.

© 2019 DeRoos LJ et al. *JAMA Network Open*

eFigure 3. Expected Number of Unique Candidates on a Solid-Organ Transplant Waiting List From 2004-2014 Associated With a 5% Increase in Donors With Presumed Consent Using Random and Ideal Allocations

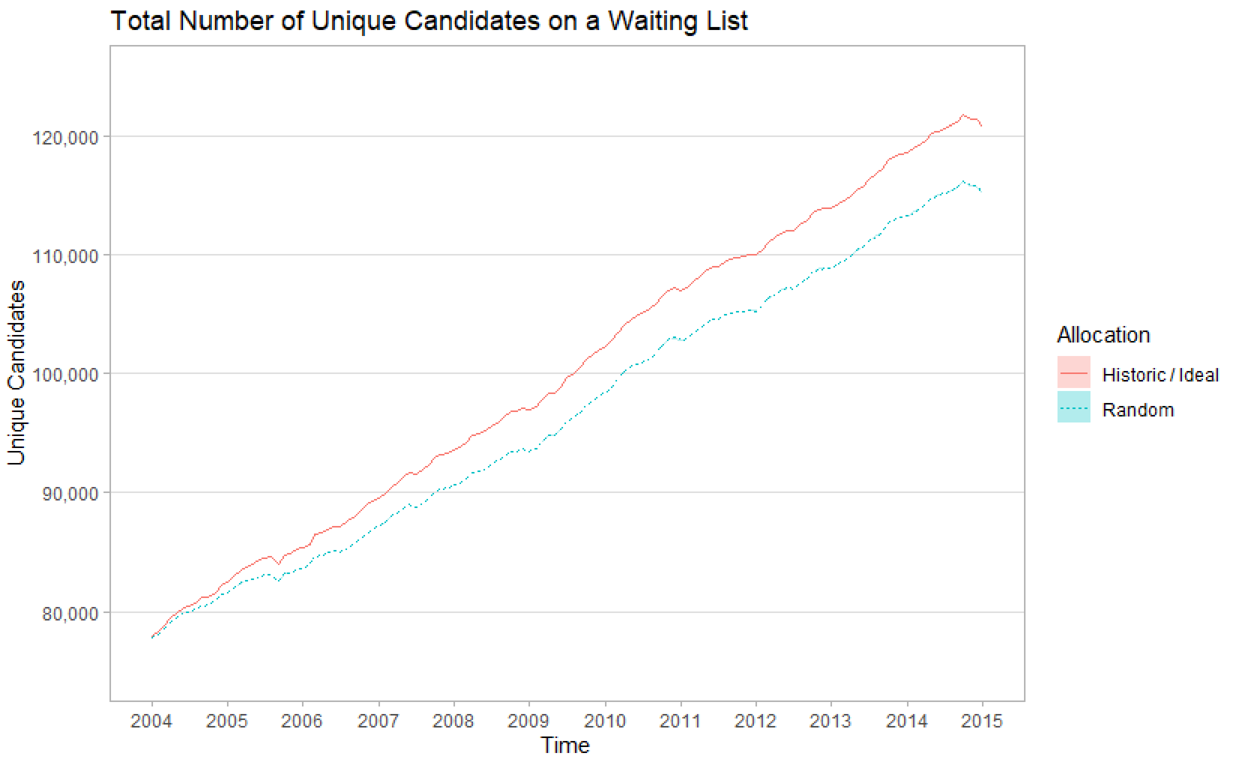

The random and ideal allocation policy estimates are assumed to be lower and upper bounds for a practical allocation policy. The true mean percent reduction in removals due to death or illness is expected to be between these allocation policy estimates. The ranges represent a non-parametric 95% confidence interval on the policy estimates due to variation in organ yield. The ideal allocation results did not differ from historical values and are plotted on the same line.

© 2019 DeRoos LJ et al. *JAMA Network Open*

eFigure 4. Expected Number of Unique Candidates on a Solid-Organ Transplant Waiting List From 2004-2014 Associated With a Range of Increases in Donors With Presumed Consent Using Random Allocation

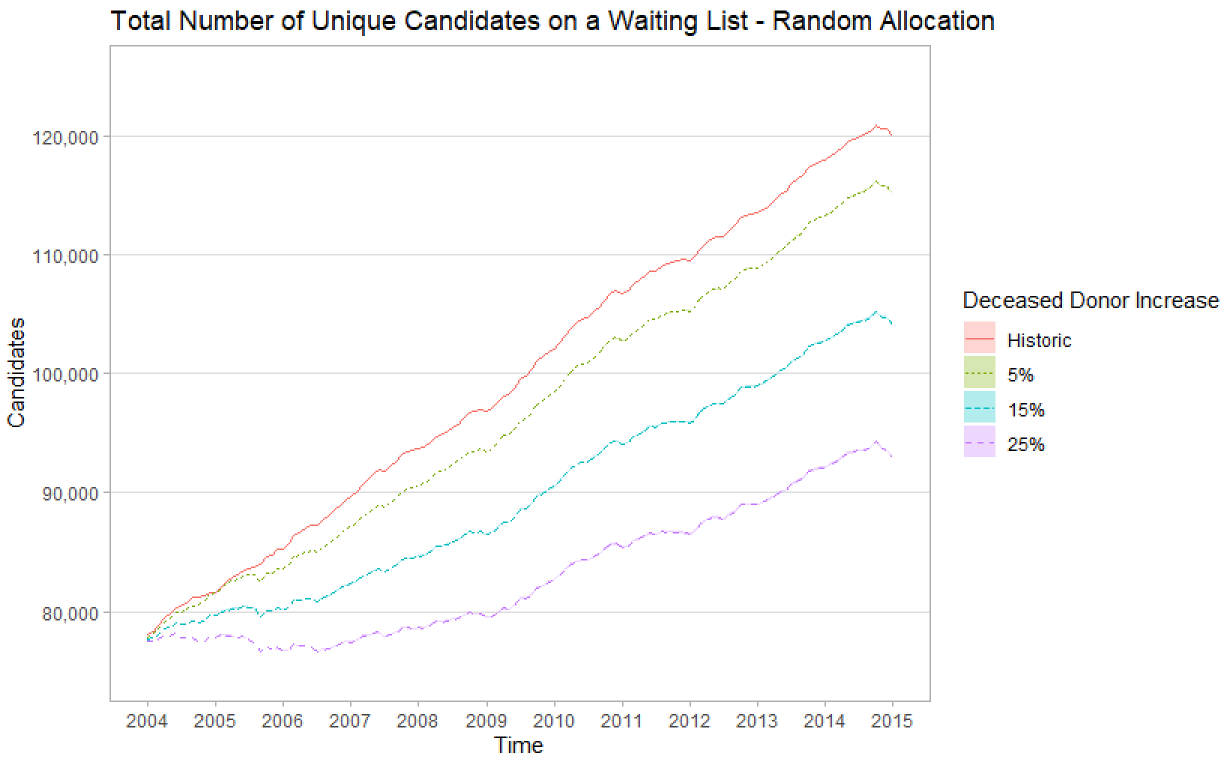

The random and ideal allocation policy estimates are assumed to be lower and upper bounds for a practical allocation policy. The ideal allocation policy results did not differ from historical values for any donor percent increase in the 5-25% donor increase range studied. The true mean percent reduction in removals due to death or illness is expected to be between the historical values and the random allocation policy estimates. The ranges represent a non-parametric 95% confidence interval on the policy estimates due to variation in organ yield.

© 2019 DeRoos LJ et al. *JAMA Network Open*

eFigure 5. Expected Number of Unique Candidates on a Solid-Organ Transplant Waiting List From 2004-2014 Associated With a 5% Increase in Donors With Presumed Consent Using Random Allocation by Organ: a) Heart; b) Kidney; c) Liver; d) Lung; e) Pancreas

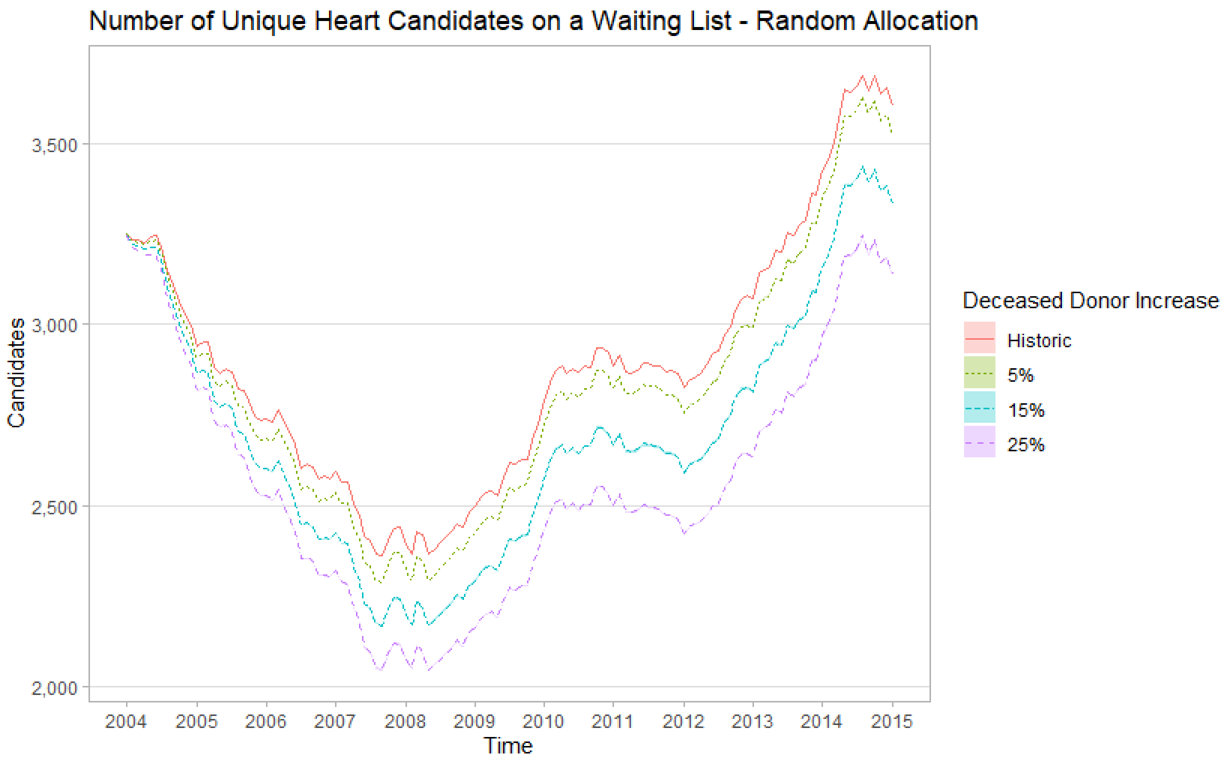

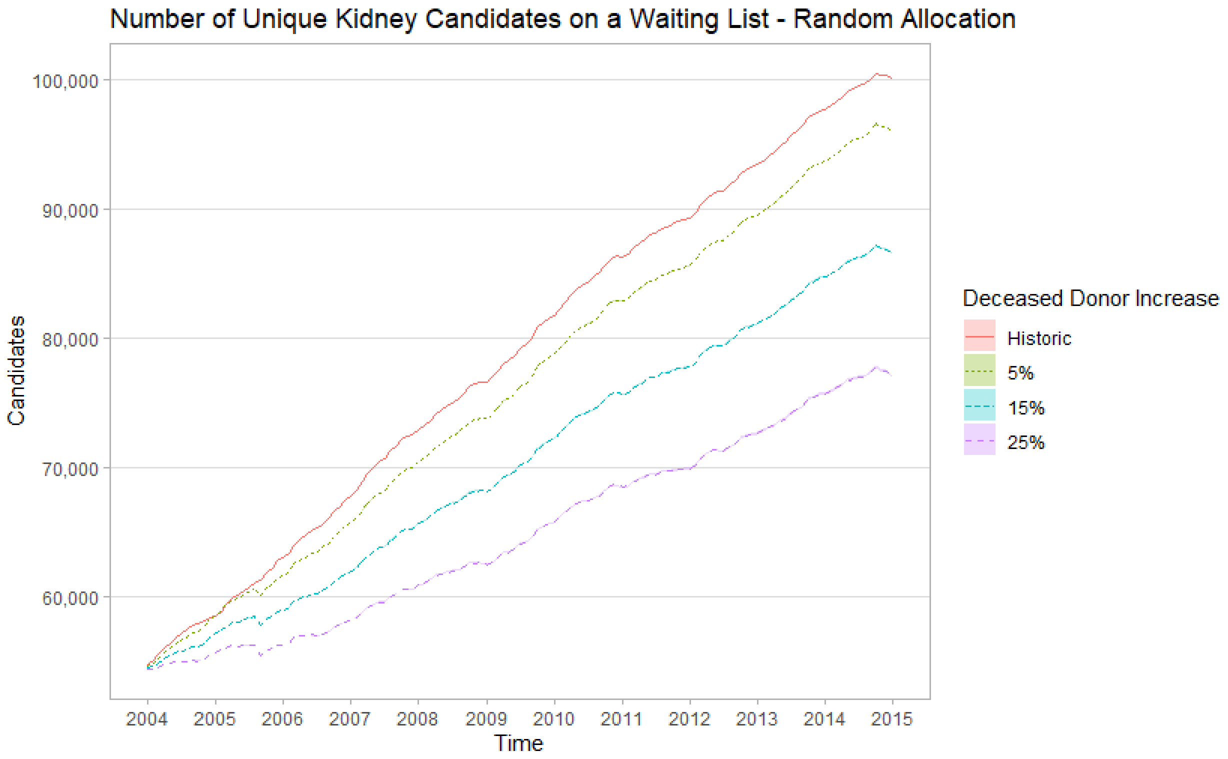

© 2019 DeRoos LJ et al. *JAMA Network Open*

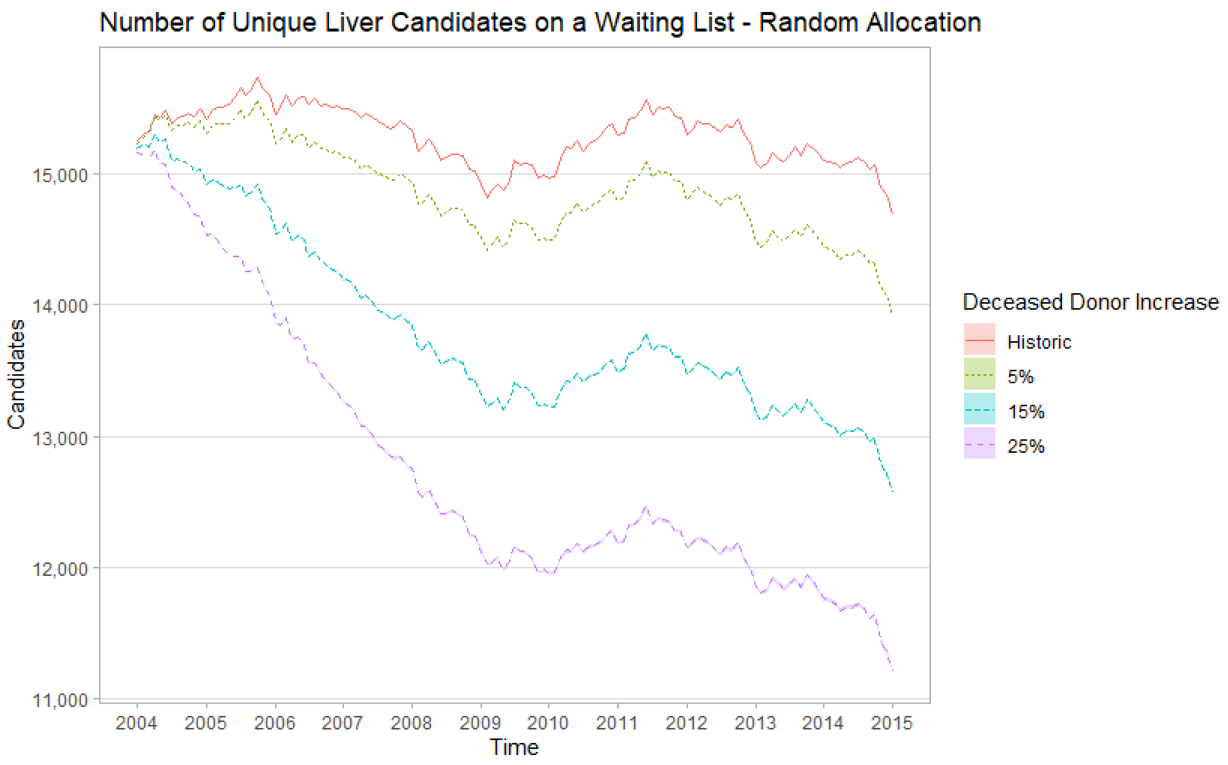

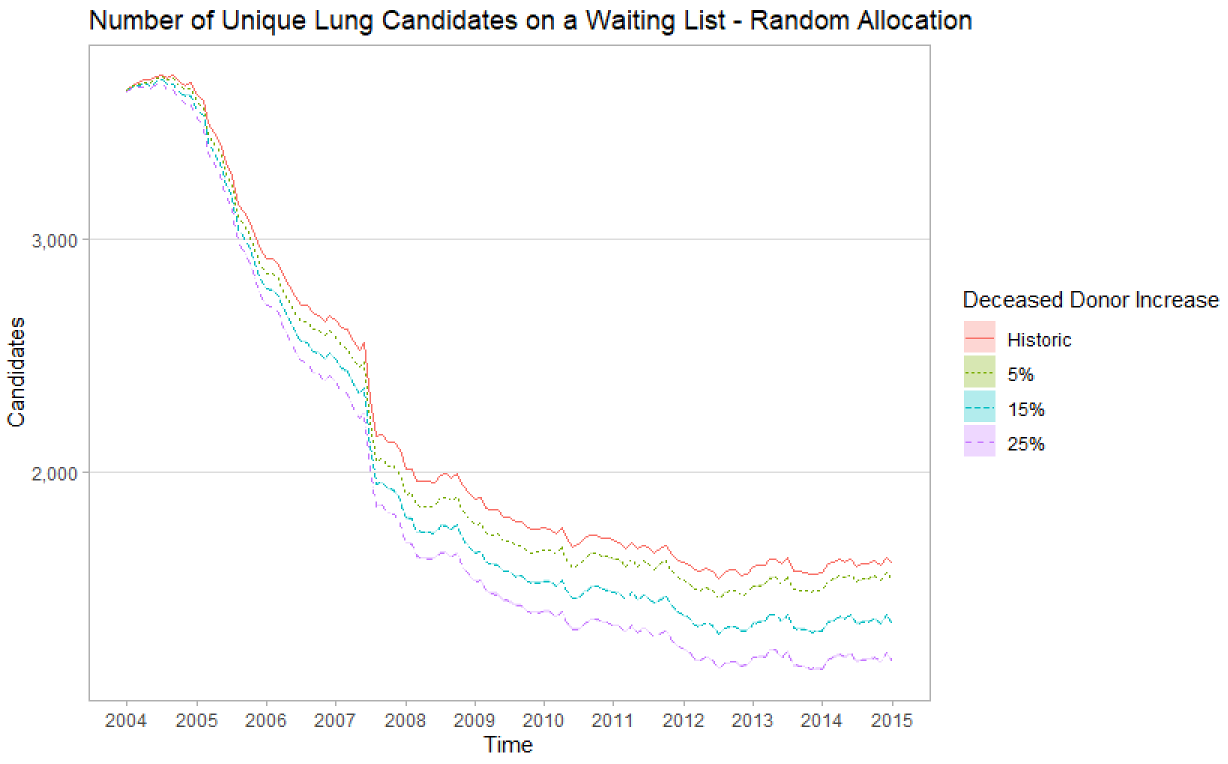

© 2019 DeRoos LJ et al. *JAMA Network Open*

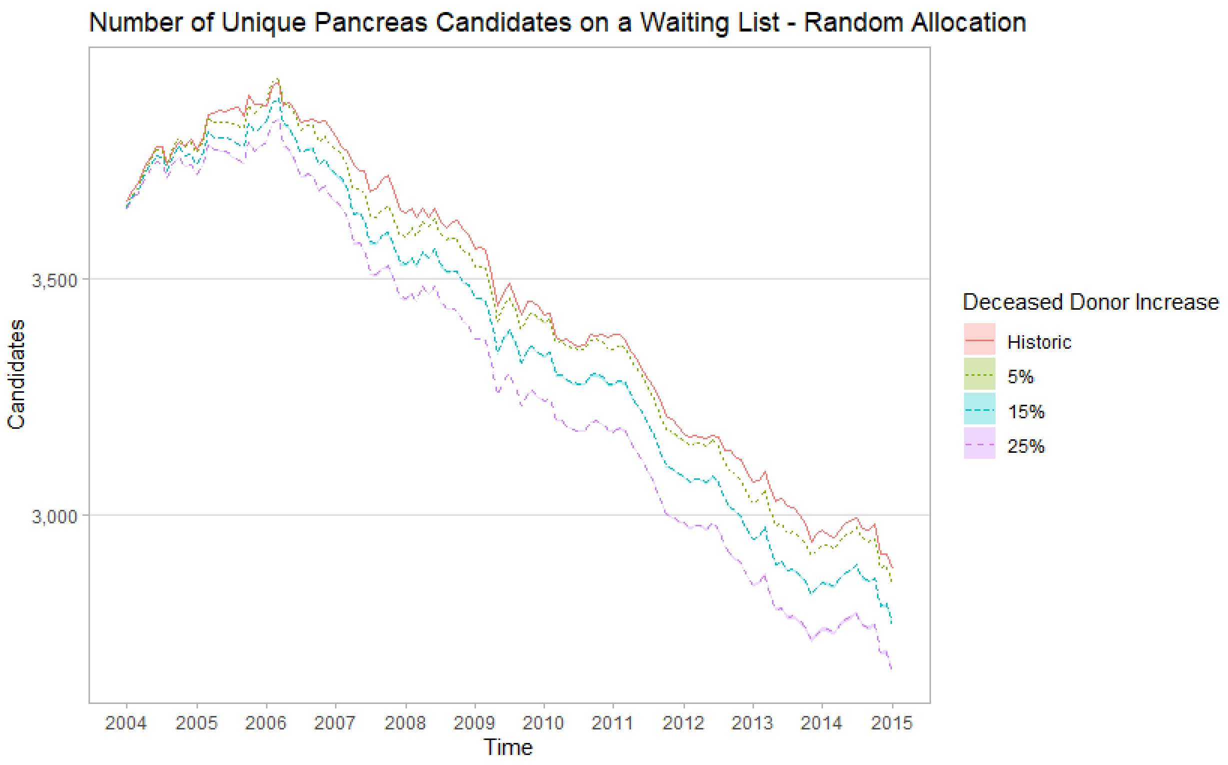

The random and ideal allocation policy estimates are assumed to be lower and upper bounds for a practical allocation policy. The ideal allocation policy results did not differ from historical values for any donor percent increase in the 5-25% donor increase range studied. The true mean percent reduction in removals due to death or illness is expected to be between the historical values and the random allocation policy estimates. The ranges represent a non-parametric 95% confidence interval on the policy estimates due to variation in organ yield.

© 2019 DeRoos LJ et al. *JAMA Network Open*
